# Water-Induced Confinement of Perfluorinated Pollutants in Biobased Polyamide Nanofibrous Membranes

**DOI:** 10.1021/acsami.5c22145

**Published:** 2026-03-18

**Authors:** Xiang Ding, Muhammad Kamran, Garyfalia A. Zoumpouli, Guadalupe Jiménez-Serratos, Carmelo Herdes, Matthew G. Davidson, Hannah S. Leese

**Affiliations:** † Department of Chemistry, 1555 University of Bath, Bath BA27AY, U.K.; ‡ Institute of Sustainability and Climate Change, University of Bath, Bath BA27AY, U.K.; § Department of Chemical Engineering, University of Bath, Bath BA27AY, U.K.; ∥ The Hartree Centre, STFC Daresbury Laboratory, Warrington WA44AD, U.K.; ⊥ School of Chemistry, 8797 University College Dublin, Dublin D04 V1W8, Ireland

**Keywords:** perfluorooctanoic acid, per- and polyfluoroalkyl substances, biobased polyamide, nanofiber membranes, regeneration, remediation, water treatment

## Abstract

Perfluorooctanoic acid (PFOA), a representative per- and polyfluoroalkyl substance (PFAS), is a persistent water contaminant due to its strong C–F bonds and amphiphilic molecular nature. Here, we reveal a water-mediated adsorption mechanism in biobased poly­(hexamethylene 2,5-furandicarboxylamide) (PA6F) nanofiber membranes, in which hydration induces structural densification and molecular confinement of PFOA within the fibrous network. Upon water exposure, the electrospun PA6F membrane undergoes macroscopic shrinkage driven by swelling and partial fusion of individual nanofibers, leading to a denser polymer matrix. This transformation promotes strong PFOA retention through a combination of hydrogen bonding, electrostatic interactions, and physical confinement, as supported by molecular dynamics simulations. The PA6F nanofiber membranes achieve a PFOA removal efficiency of 94.6% and an adsorption capacity of 3.92 mg g^–1^ at industrially polluting concentrations. Thermal regeneration at 240 °C enables complete release of confined PFOA while preserving the polymer backbone. The recovered polymer can be reprocessed by re-electrospinning to form new nanofiber membranes that retain 93% of the original adsorption capacity after reuse. These findings provide water-mediated confinement mechanisms in more sustainable polyamide systems, establishing a closed-loop adsorption-regeneration pathway for long-term PFAS remediation in aqueous environments.

## Graphical abstract

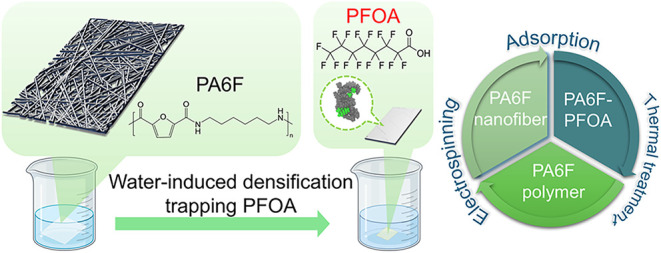

## 1 Introduction

Perfluorooctanoic acid (PFOA), a representative member of the per- and polyfluoroalkyl substance (PFAS) family, has been extensively used in industries such as fluoropolymer manufacturing, textiles, firefighting foams, and nonstick coatings due to its thermal stability, hydrophobicity and chemical resistance.^1,2^ However, its environmental persistence, bioaccumulative potential, and toxicity have raised significant concerns globally.^3,4^ Long-term exposure to PFOA has been associated with increased risks of cancer, endocrine disruption, and adverse effects on the immune system, driving efforts to develop effective methods for its removal from water sources.^5,6^

Various strategies have been developed to mitigate PFAS contamination in water, including adsorption,^7,8^ electrochemical oxidation,^9^ photocatalysis,^10^ and bioremediation.^11^ Among these, adsorption remains particularly attractive due to its operational simplicity, scalability, and reduced energy input.^12,13^ Traditional adsorbents such as granular activated carbon (GAC), ion-exchange resins, and metal oxides have been widely used for PFOA removal.^14–16^ Although GAC and ion-exchange resins exhibit a strong affinity toward long-chain PFAS, they face challenges such as slow kinetics, limited regeneration capability, and maintenance requirements that hinder widespread application in large-scale industrial systems. In recent years, various alternative adsorbent materials have been explored, such as molecularly imprinted polymers,^17^ graphene oxide-based composites,^18^ and metal–organic frameworks,^19^ which have shown promising selectivity and high adsorption capacities. Despite these advances, many of these systems rely on complex synthesis routes, nonrenewable feedstocks, or costly raw materials, and often suffer from limited recyclability. These limitations present significant challenges for long-term and large-scale deployment, particularly in the context of sustainable water treatment.

The challenge is further compounded by the wide range of PFOA concentrations encountered in industrial wastewater. While environmental levels are often in the parts-per-trillion range, effluents from specific industrial processes, such as microelectronics manufacturing, can contain PFOA at concentrations as high as 3.35 mM.^20^ This range places greater demands on the adsorption capacity and durability of the materials used. Therefore, the development of cost-effective, sustainable, and regenerative adsorbents for PFOA removal from industrial effluents remains a critical need.

Recent advances in polymer chemistry have enabled the design of biobased semiaromatic polyamides that integrate renewable furanic units, combining the chemical robustness of conventional nylons with enhanced polarity and hydrogen-bonding capability.^21,22^ Poly­(hexamethylene 2,5-furandicarboxylamide) (PA6F), synthesized from renewable monomers, exhibits high thermal stability and strong intermolecular interactions arising from its furan ring and amide groups.^23,24^ Although its bulk properties have been reported, the interfacial behavior of PA6F in aqueous environments and its potential role in pollutant adsorption mechanisms remain unexplored.

Electrospun nanofiber membranes have emerged as attractive platforms for water treatment applications due to their high surface-area-to-volume ratio, interconnected porous networks, and tunable surface chemistry.^25,26^ Their fibrous architecture allows rapid mass transfer and enhanced molecular interactions, which can significantly enhance adsorption performance.^27^

In this work, we introduce a sustainable sorbent platform based on electrospun nanofiber membranes of the bioderived semiaromatic polyamide PA6F. Upon water exposure, the PA6F nanofibers undergo an unusual swell-shrink transition in which individual fibers swell and partially fuse, leading to macroscopic membrane contraction and matrix densification. This water-triggered structural transformation generates molecular confinement domains that strongly retain PFOA through coupled hydrogen-bonding and electrostatic interactions, as supported by molecular dynamics simulations. Compared with structurally related polyamides, nylon-6 (PA6) and nylon-66 (PA66), the electrospun PA6F membranes exhibit markedly enhanced adsorption kinetics and capacity, highlighting the critical role of the furan-containing backbone and water-induced matrix reorganization. Furthermore, the PA6F nanofibrous membranes can be regenerated through mild and controlled thermal treatment to remove adsorbed PFOA while preserving the polymer backbone. The regenerated material can be redissolved and re-electrospun to form new nanofiber membranes that maintain comparable adsorption performance on reuse. These findings uncover a previously unrecognized water-induced confinement process in polyamides that governs PFAS retention and release, offering new molecular-level insight into the adsorption-regeneration pathways of persistent pollutants in polymeric systems.

## 2 Materials and Methods

### 2.1 Materials

Poly­(hexamethylene furanamide) (PA6F) was synthesized following a previously reported method using dimethyl 2,5–furandicarboxylate (DMFDC) and hexamethylenediamine (HMDA).^23,24^ The resulting polymer was purified and dried prior to use. Nylon-6 (PA6), nylon-66 (PA66), formic acid (≥98%), and dichloromethane (DCM, ≥99.8%) were purchased from Sigma-Aldrich. Perfluorooctanoic acid (PFOA), used as the target organic pollutant in this study, and ammonium acetate were also obtained from Sigma-Aldrich. The native analytical standard of PFOA and its corresponding stable-isotope-labeled internal standard (used for quantification) were supplied by Greyhound Chromatography. Ultrapure water (18.2 MΩ), produced via a Veolia Purelab Chorus system, was utilized in preparing all solutions. All reagents and solvents were used as received.

### 2.2 Preparation of Electrospun Nanofiber Membrane

PA6F nanofiber membranes were prepared via electrospinning. Briefly, PA6F was dissolved in a binary solvent system of formic acid and dichloromethane (1:1 v/v) to prepare a 30% w/v homogeneous polymer solution. Electrospinning was performed using a single-needle setup equipped with a rotating drum collector covered with silicone-coated parchment paper. Electrospinning was conducted under ambient conditions (22–25 °C, relative humidity 30–40%). After collection, the nanofiber membranes were dried under vacuum at 50 °C overnight to remove residual solvent prior to characterization and use in adsorption experiments.

For comparison, PA6 and PA66 nanofiber membranes were also prepared using a similar binary solvent system and electrospinning procedure.

### 2.3 Characterization of PA6F Electrospun Membranes

The morphology of the electrospun nanofibers was observed using a scanning electron microscope (Hitachi, SU3900) operating at an accelerating voltage of 10 kV. Prior to imaging, each sample was sputtered with a 20 nm thick gold layer using a sputter coater (Quorum Technologies, Q150TS).

Pore size and pore size distribution of the PA6F nanofiber membranes were analyzed using a gas liquid porometer (POROLUX 1000). Prior to measurement, dry membrane samples were fully wetted with the standard wetting liquid POREFILL (POROMETER NV, Belgium), and the mean flow pore size was recorded to represent the average effective pore diameter.

The specific surface area was determined by nitrogen adsorption using the Brunauer–Emmett–Teller (BET) method. Prior to measurement, all dried samples were degassed at 50 °C under vacuum for 18 h to remove physically adsorbed moisture without inducing thermal or structural changes in the polymer. Measurements were performed on a 3Flex physisorption analyzer (Micromeritics) and nitrogen adsorption–desorption isotherms were recorded at 77 K.

Surface topography and nanoscale roughness were further characterized by atomic force microscopy (AFM, Oxford Instruments Jupiter XR) in tapping mode. Height images were processed using Gwyddion software to calculate root-mean-square (RMS) roughness values.

Fourier-transform infrared (FTIR) spectra were recorded using a Bruker INVENIO spectrometer. Spectra were collected in the range of 4000–500 cm^–1^ at a resolution of 4 cm^–1^. Prior to analysis, all samples were dried at 50 °C under vacuum to remove residual solvent and moisture. Dried membrane samples were measured directly in the solid state without further treatment.

X-ray diffraction (XRD) analysis was conducted in transmission mode with Cu Kα radiation (λ = 1.5406 Å), operating at 40 kV and 40 mA. Scans were recorded in the 2θ range of 2–75° to analyze the crystalline structure of the membranes. Electrospun membranes were dried prior to analysis and mounted directly onto the sample holder without additional processing.

Water contact angle (WCA) was measured using a contact angle goniometer (Dataphysics OCA 25) by placing a 3 μL droplet of deionized water on the membrane surface, and the results were analyzed using SCA 20 software.

Water uptake measurements were performed by immersing preweighed membrane samples (3 cm × 3 cm) in deionized water at room temperature for 1 h. The membranes were then gently blotted to remove surface water and weighed again. Water uptake was calculated using the following equation

$$\mathrm{water}\;\mathrm{uptake}\;\left(\%\right)=\frac{W_{\mathrm{wet}}-W_{\mathrm{dry}}}{W_{\mathrm{dry}}}\times 100$$

Where *W*_wet_ and *W*_dry_ are the weights of the wet and dry membranes, respectively.

To evaluate shrinkage behavior, membrane samples (3 cm × 3 cm) were placed in individual Petri dishes and gently submerged in 10 mL of deionized water to avoid folding or disturbance. After 1 or 24 h of immersion, the water was removed using a pipet. Samples were then left in a fume hood for drying. Images were taken before water exposure and after drying, and the change in membrane area was analyzed using ImageJ software to calculate the percentage shrinkage.

X-ray photoelectron spectroscopy (XPS, Kratos Axis SUPRA) was used to analyze the surface elemental composition and chemical states before and after PFOA adsorption. Survey and high–resolution scans (C 1s, N 1s, and O 1s) were acquired with a monochromatic Al Kα source (1486.6 eV). Membrane samples were thoroughly dried before analysis and measured directly in their solid state.

### 2.4 PFOA Adsorption Experiments

The adsorption performance of PA6F nanofiber membranes toward perfluorooctanoic acid (PFOA) was evaluated through batch adsorption experiments under ambient conditions. Ten mg membrane samples were immersed in 10 mL of PFOA solution with a known initial concentration (typically 10 μM unless otherwise stated). All batch adsorption experiments were performed in triplicate unless otherwise stated. Blank control experiments without PA6F membrane were conducted under identical conditions to account for potential nonspecific losses of PFOA due to adsorption to container walls or other effects.

PFOA concentrations were determined using high-performance liquid chromatography coupled with single quadrupole mass spectrometry (HPLC-MS, Agilent 1260 Infinity II). At predetermined time intervals, an aliquot (5 μL) was withdrawn from the adsorption solution and diluted prior to analysis. LC-MS samples were prepared by mixing 5 μL of the withdrawn aliquot with 25 μL of an isotopically labeled internal standard solution and 470 μL of ultrapure water to obtain a final volume of 500 μL. The internal standard was therefore added during sample preparation before instrumental analysis. Further details are provided in the Supporting Information. PFOA removal efficiency was evaluated by plotting the normalized concentration [C]/[C_0_] as a function of time. The equilibrium adsorption capacity of PA6F nanofiber membranes was determined using the following equation

$$q_{e}=\left(C_{0}-C_{e}\right)\times V/M$$

where *C*_0_ and *C*_e_ are the initial and equilibrium concentrations of PFOA, *V* is the volume of the solution, and *M* is the mass of the nanofiber membrane.

### 2.5 Reusability Study

Thermogravimetric-mass spectrometric (TGA-MS) analysis was carried out using a simultaneous thermal analyzer (NETZSCH STA 449 F1) coupled with a quadrupole mass spectrometer (QMS 403C Aeolos). Samples (∼10 mg) were heated from 30 to 600 °C at a rate of 10 °C/min under nitrogen flow (50 mL/min). The mass spectrometer recorded signals in the *m*/*z* range of 15–300.

The reusability of PA6F nanofiber membranes was evaluated by subjecting PFOA-loaded membranes to thermal treatment, followed by polymer recovery and re-electrospinning. After adsorption saturation, membranes were dried under vacuum at 50 °C. The dried membranes were then heated at 240 °C for 4 h under a nitrogen atmosphere in a tubular furnace. Following thermal treatment, the resulting residues were collected and dissolved in the same binary solvent system (formic acid/DCM, 1:1 v/v) to prepare a new spinning solution. The recovered polymer was then electrospun under previously optimized conditions to produce regenerated nanofiber membranes.

### 2.6 Molecular Dynamics Simulations

All-atom molecular dynamics simulations were performed using GROMACS^28–30^ and the GROMOS54A7 force field, with parameters derived from the Automated Topology Builder (ATB)^31^ for PA6F, PFOA, and SPC water. A typical system included 6 PA6F oligomers (*n* = 5), 12 PFOA molecules, and 32,000 SPC water molecules in a cubic box with periodic boundary conditions. After energy minimization, the system was equilibrated in NPT ensemble for 1 ns at 300 K, followed by 20 ns production run. Radial distribution functions (*g*(*r*)) were computed between PFOA, polymer and water.

## 3 Results and Discussion

### 3.1 Dimensional Transformation and Shrinkage Behavior of PA6F Nanofiber Membranes

Poly­(hexamethylene 2,5-furandicarboxylamide) (PA6F) was fabricated into nanofibrous membranes via electrospinning using a single-spinneret configuration (Figure 1a). Following a design-of-experiments (DOE) optimization process, continuous and bead-free PA6F nanofibers with good morphological uniformity were obtained under the optimized conditions (Tables S1–S3 and Figures S1 and S2, Supporting Information). As shown in Figure 1b, the resulting nanofibers exhibit an average diameter of 141 ± 37 nm. Fourier transform infrared (FTIR) spectroscopy was performed to compare the polymer powder and electrospun nanofibers, confirming the absence of residual solvent in the PA6F membranes (Figure S3, Supporting Information).

**Figure 1:**
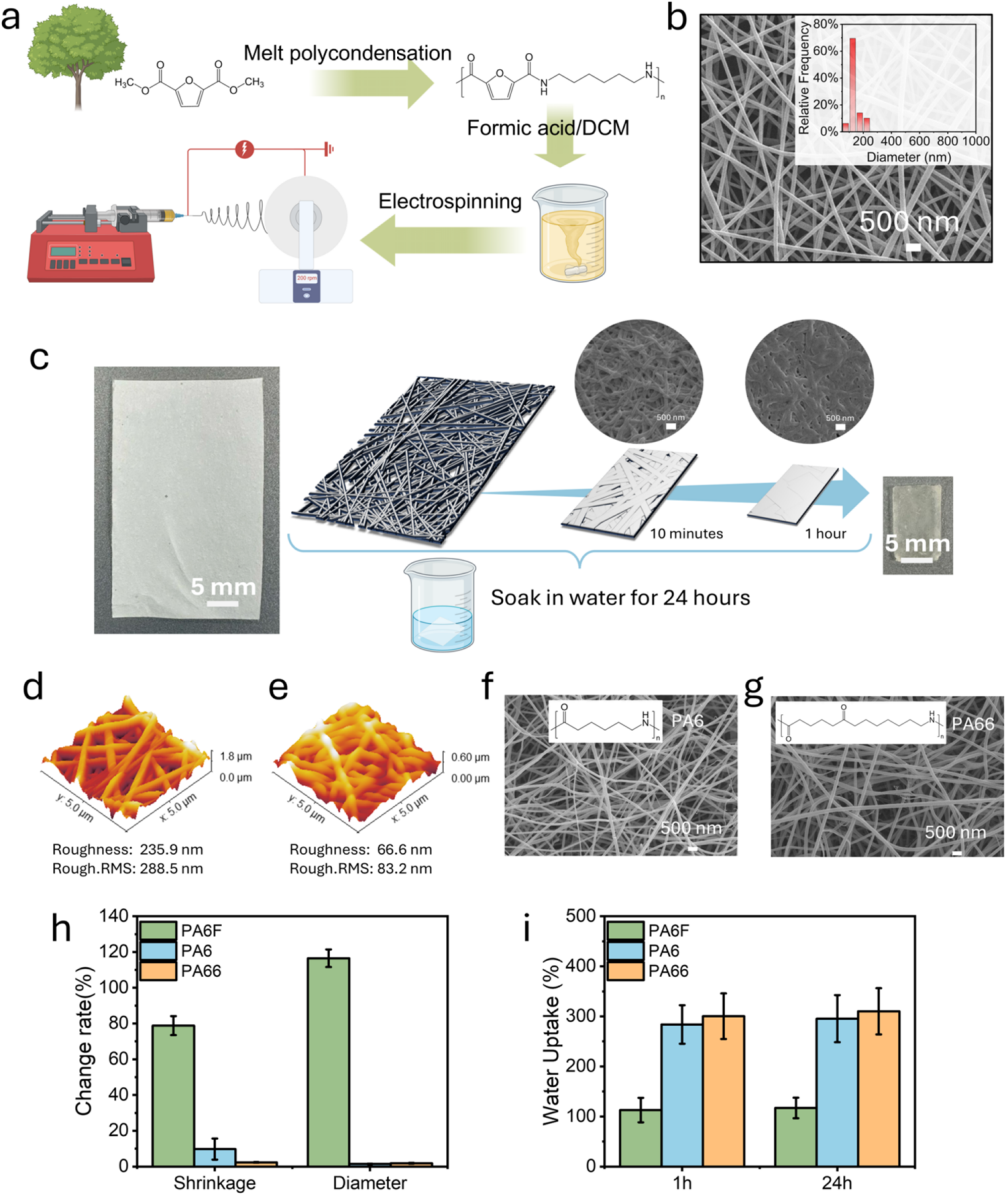
Swelling-shrinkage behavior and water uptake performance of PA6F nanofiber membranes compared to PA6 and PA66. (a) Schematic of the fabrication process of electrospun poly­(hexamethylene 2,5-furandicarboxylamide), PA6F, nanofiber membranes. (b) SEM image of the electrospun PA6F nanofibers and the corresponding fiber diameter distribution. (c) Schematic of the coupled macroscopic shrinkage and microscopic swelling behavior of PA6F nanofiber membranes during water immersion. (d, e) AFM images of PA6F nanofibers (d) before and (e) after 1 h of water immersion. (f, g) SEM images of electrospun (f) PA6 and (g) PA66 nanofiber membranes with uniform fiber morphology. (h) Comparison of area shrinkage and fiber diameter changes after 1 h of immersion for PA6F, PA6, and PA66 membranes. (i) Water uptake of PA6F, PA6, and PA66 nanofiber membranes after 1 and 24 h of immersion.

A unique characteristic observed in the electrospun PA6F nanofiber membranes was their dynamic swell-shrink behavior upon immersion in water. Macroscopically, the membrane underwent significant area contraction over time when submerged (Figure 1c). This shrinkage originates from swelling of individual nanofibers, which increases their diameters and brings them into closer contact with each other. As swelling progresses, interfiber fusion occurs, reducing the overall porosity and flexibility of the network. The combined loss of internal void space and restricted chain mobility ultimately drives the observed macroscopic shrinkage of the membrane (Figure 1c).

Atomic force microscopy provided further insight into the nanoscale morphological evolution of the PA6F fibers. The fiber surfaces became smoother and more compact after 1 h of swelling in water (Figure 1d, before swelling, Figure 1e, after swelling), also confirmed by the reduction in root-mean-square surface roughness from 288.5 to 83.2 nm. These observations indicate that fiber fusion and plasticization occurred during hydration, contributing to the loss of individual fiber boundaries and leading to macroscopic shrinkage.

To quantitatively assess the porous characteristics of the electrospun PA6F nanofiber membranes, nitrogen adsorption (BET) and capillary flow porometry measurements were performed (Table S4 and Figure S4, Supporting Information). The pristine, dry PA6F membrane exhibited a surface area of 15.6 m^2^/g, consistent with its nanofibrous and highly porous morphology. Capillary flow porometry revealed a mean flow pore (MFP) size of 0.43 μm, confirming the presence of interconnected micron-scale pores within the membrane network. Upon immersion in water after 1 h, the measured BET surface area decreased sharply to 0.0095 m^2^/g, and no reliable BET measurement could be obtained after 24 h of immersion. This pronounced loss of accessible surface area reflects extensive fiber swelling, pore collapse, and interfiber fusion, in agreement with SEM and AFM observations.

To assess whether this behavior, for which we know no precedent, was common across other similar polyamides, we investigated PA6 and PA66. Both polymers share aliphatic amide linkages with PA6F, and all three materials have repeating −CONH– groups along their backbone. The key distinction lies in the incorporation of an aromatic furan ring in PA6F, in contrast to the fully aliphatic structure of PA6 and PA66. Using a similar binary solvent system and optimized electrospinning conditions, uniform nanofiber membranes of PA6 (Figure 1f) and PA66 (Figure 1g) were successfully fabricated. The average fiber diameters were 136 ± 36 nm for PA6 and 158 ± 23 nm for PA66, comparable to that of PA6F (141 ± 37 nm).

Despite their similar morphology, the swelling behavior of PA6 and PA66 membranes diverged significantly from that of PA6F. Upon the same water immersion treatment, PA6F membranes exhibited a pronounced shrinkage of 78.8 ± 5.1%, whereas PA6 and PA66 membranes displayed a slight area shrinkage of 9.8 ± 5.9% and 2.3 ± 0.2%, respectively (Figure 1h). After water immersion, SEM images showed that the fibers of PA6 and PA66 remained distinct and spatially separated, without fusion or structural collapse (Figure S5, Supporting Information), in stark contrast to PA6F.

Quantitative analysis of fiber diameter changes further supports this observation. After 1 h of water immersion, the average fiber diameter of PA6F increased by 116%, indicating substantial swelling at the individual nanofiber level. In comparison, the diameter change in PA6 and PA66 fibers was minimal, remaining below 2%, suggesting limited water penetration or chain expansion.

Although PA6F showed dramatic morphological changes in nanofiber swelling, its water uptake was 112.86%, considerably lower than that of PA6 (283.81%) and PA66 (300.37%) after 1 h of immersion (Figure 1i). This result suggests that water absorbed by PA6F is more localized within the fiber matrix and is likely associated within the polymer network through specific polymer–water interactions, rather than existing as freely retained bulk water.

### 3.2 Water-Induced Changes in Surface Properties and Mechanical Behavior

To further understand the unusual water-responsiveness of PA6F nanofiber membranes, the surface chemistry, wettability, and mechanical behavior were investigated before and after water exposure.

FTIR spectroscopy was carried out to investigate the molecular interactions between PA6F and water during swelling (Figure 2a). After 1 h of water immersion, noticeable changes were observed in the FTIR spectra of the PA6F membrane (PA6F-w). In particular, the N–H stretching band around 3295 cm^–1^ exhibited clear broadening, consistent with enhanced hydrogen-bond-related interactions involving the amide groups upon water uptake. More specifically, the amide I band (C=O stretching) shifted from 1647 to 1644 cm^–1^, and the amide II band (N–H bending and C–N stretching) shifted from 1538 to 1533 cm^–1^, reflecting changes in the local chemical environment of the amide functionalities. These shifts are characteristic of hydrogen bond formation between water molecules and the carbonyl and amine groups in the polyamide backbone.^32^ The overall reduction in intensity and subtle band shifts further suggest increased molecular mobility and partial chain rearrangement, consistent with the observed macroscopic swelling and matrix densification. In contrast, PA6 and PA66 membranes exhibited negligible spectral changes after water immersion followed by drying (Figure S6, Supporting Information). The amide I and II bands retained their original positions and intensities, suggesting limited hydrogen bonding with water and no significant structural rearrangement during the treatment.

**Figure 2:**
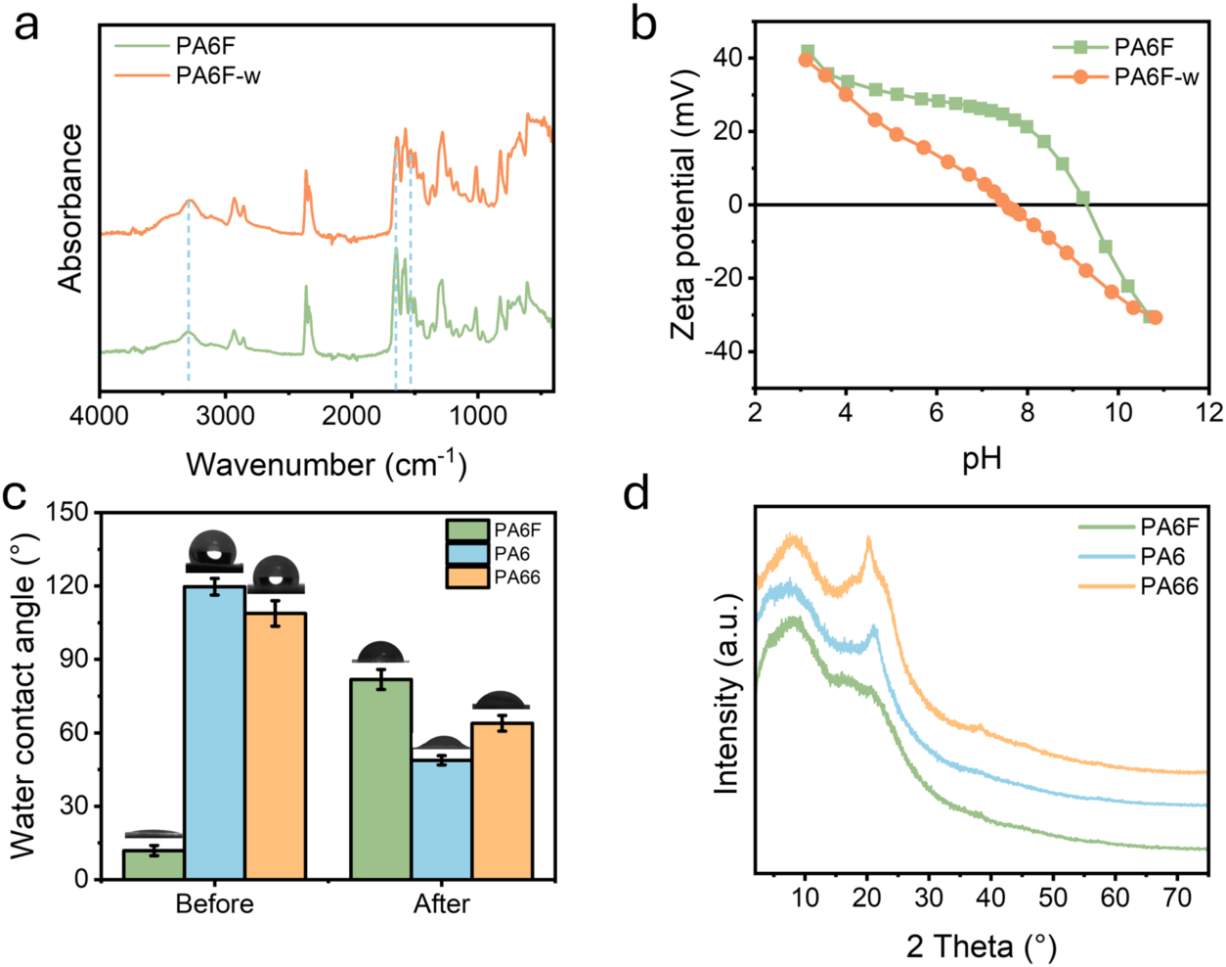
Structural and surface property changes of PA6F nanofiber membranes upon water exposure. (a) FTIR spectra and (b) Zeta potential profiles of PA6F nanofiber membranes before (PA6F) and after (PA6F-w) 1 h of water immersion. (c) Water contact angle measurements of PA6F, PA6, and PA66 membranes before and after soaking in water. (d) XRD patterns of PA6F, PA6, and PA66 membranes.

Zeta potential measurements were conducted to probe changes in the interfacial charge characteristics of PA6F nanofibrous membranes in aqueous environments (Figure 2b). It should be noted that zeta potential measurements are performed under fully hydrated conditions. In this context, the distinction between “PA6F” and “PA6F-w” reflects differences in the water-conditioning history of the membranes prior to measurement, rather than differences in hydration state during the experiment itself. Specifically, PA6F-w membranes were immersed in water for 1 h and subsequently air-dried at room temperature before zeta potential analysis. This preconditioning treatment induces pronounced fiber swelling followed by partial interfiber fusion and irreversible matrix densification, which persist upon reimmersion during the measurement.

The pristine PA6F membrane exhibited a moderately positive zeta potential over the pH range of 3–9, with an isoelectric point (IEP) at approximately pH 9.2. Following water conditioning, the zeta potential profile shifted systematically, and the IEP decreased to around pH 7.4. The observed shift in zeta potential and isoelectric point following water conditioning is attributed to hydration-induced reorientation and redistribution of polar amide functionalities and interfacial dipoles within the PA6F matrix, which alters the effective charge environment at the shear plane. Importantly, this behavior does not imply the formation or exposure of new acidic surface groups but instead reflects changes in the spatial arrangement and accessibility of existing polar groups under hydrated conditions.

Water contact angle (WCA) measurements revealed significant differences in initial surface wettability and its evolution after immersion (Figure 2c). Pristine PA6F nanofiber membranes exhibited a very low water contact angle of 12°, confirming their highly hydrophilic surface due to the presence of accessible amide groups and polar furan rings. PA6 and PA66 membranes initially displayed much higher water contact angles of 120° and 109°, respectively, indicating more hydrophobic surface characteristics in the dry state. Water droplets rapidly penetrated all three pristine membranes within 20 s, consistent with the porous nanofiber structures and the presence of hydrophilic groups. After immersion in water for 1 h and subsequent air drying, all three membranes showed significant changes in contact angle, indicating surface rearrangement. The WCA of PA6F increased to 82°, reflecting a noticeable reduction in surface hydrophilicity, although the surface remained slightly hydrophilic. In contrast, PA6 and PA66 exhibited lower WCA of 66° and 64°, respectively, indicating enhanced surface hydrophilicity, likely due to the increased water uptake. This contrasting behavior may be attributed to the higher chain mobility of PA6 and PA66, where polar groups become more accessible upon hydration. In the case of PA6F, however, partial structural densification and chain reorganization during drying likely limit the exposure of hydrophilic functional groups on the membrane surface.^33^

X-ray diffraction (XRD) patterns (Figure 2d) reveal pronounced differences in crystallinity among the PA6F, PA6, and PA66 nanofiber membranes. PA6 and PA66 both exhibit broad but discernible peaks in the 2θ range of 20–24°, characteristic of the α-crystalline phase common to aliphatic polyamides. Among them, PA66 shows a sharper and more intense peak, indicating a higher degree of crystallinity, likely due to its more symmetrical molecular structure and extensive hydrogen bonding. In contrast, PA6F exhibits a broad, low-intensity halo, consistent with a largely amorphous structure. This lack of crystallinity suggests that PA6F has a lower packing density, which makes it more accessible to water molecules. The disordered arrangement of the polymer chains also facilitates the exposure of polar groups, such as amide and ether functionalities, promoting hydrogen bonding with water and contributing to its pronounced swelling behavior. In comparison, the highly crystalline domains in PA6 and PA66 result in a more rigid and tightly packed structure, which restricts chain mobility and limits the accessibility of functional groups, suppressing their water-swelling behavior and adsorption efficiency.

Thus, the dramatic swelling and shrinkage behavior of PA6F is attributed to a combination of its semiaromatic structure and strong polar interactions. The furan ring enhances dipole–dipole interactions with water, while the flexible hexamethylene segments facilitate chain relaxation and rearrangement during hydration.^34^ In contrast, although PA6 and PA66 are aliphatic polyamides, their highly crystalline structures impose significant physical constraints on molecular motion, which limits fiber deformation and prevents large-scale matrix collapse.

Mechanical behavior was also strongly affected by water exposure. While pristine PA6F membranes were flexible and foldable, they became visibly brittle and fragile after water treatment. Tensile testing (Figure S7, Supporting Information) confirmed this transition: the tensile strength of PA6F increased from 5.32 to 17.80 MPa, but the elongation at break dramatically decreased from 11.31% to 2.50%, indicating that while the membrane became stiffer, it also lost ductility, likely due to interfiber fusion and chain immobilization caused by swelling-induced rearrangement.

### 3.3 Perfluorooctanoic Acid (PFOA) Adsorption Performance

Given the significant surface and structural changes observed in PA6F membranes upon water exposure, their capacity to adsorb perfluorooctanoic acid (PFOA) was evaluated using Liquid Chromatography–Mass Spectrometry (LC-MS) (see calibration curve for PFOA quantification in Figure S8, Supporting Information). To simulate the PFOA concentrations typically found in industrial wastewater, a 10 μM PFOA solution was selected for all adsorption experiments. When 10 mg of the electrospun PA6F nanofiber membrane was immersed in 10 mL of 10 μM PFOA solution, rapid adsorption occurred (Figure 3a), with nearly 50% removal achieved within the first hour. After 7 h, the removal rate reached 71%, demonstrating efficient uptake kinetics.

**Figure 3:**
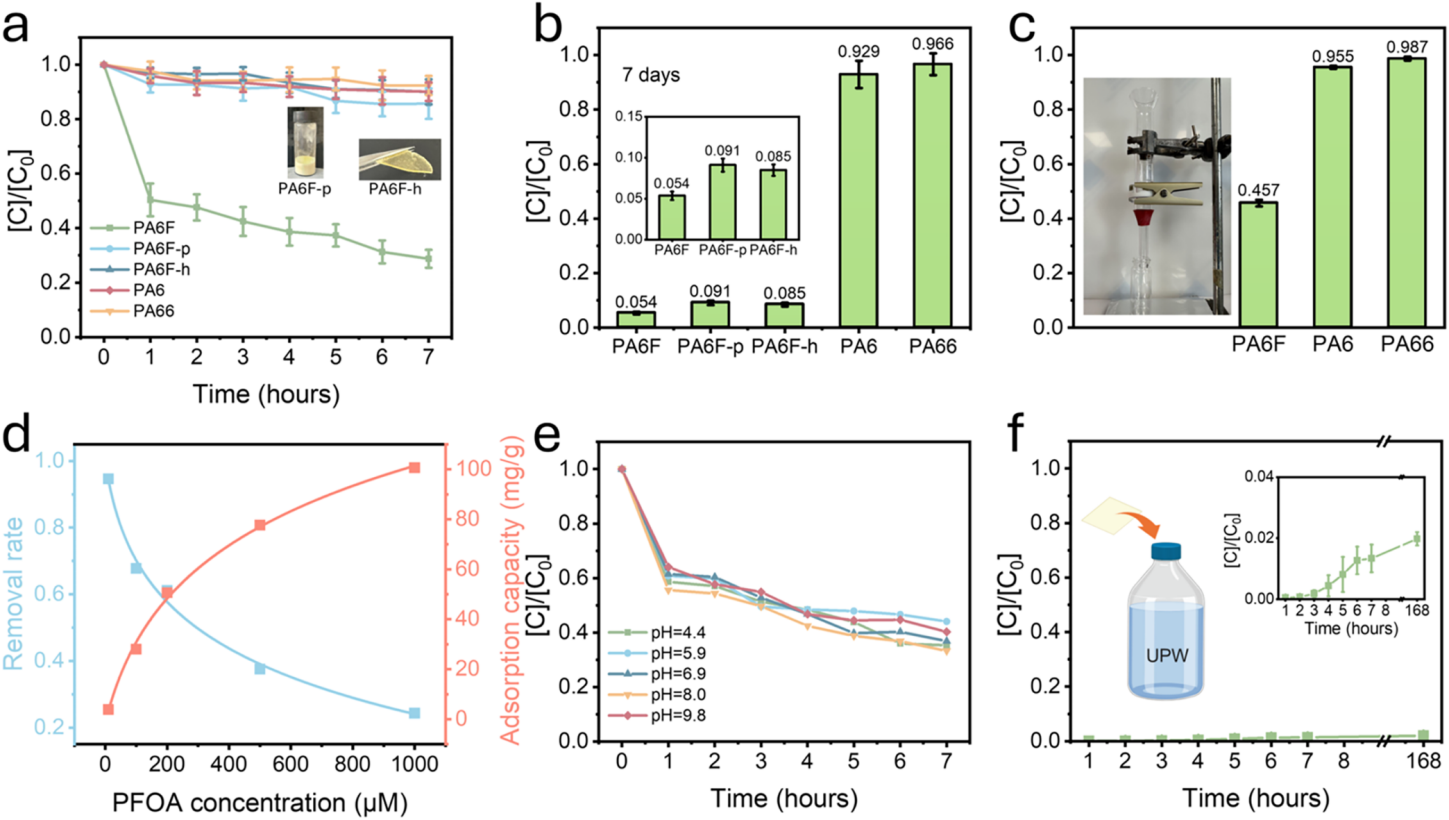
PFOA adsorption performance of PA6F, PA6, and PA66 nanofiber membranes under different conditions. (a) Time-dependent PFOA removal by electrospun PA6F nanofiber membranes (10 mg in 10 mL of 10 μM PFOA solution), compared with PA6, PA66, PA6F powder (PA6F-p), and hot-pressed PA6F film (PA6F-h). (b) Long-term adsorption of PFOA over 7 days. (c) PFOA removal efficiency of PA6F membrane in a gravity-driven filtration setup. (d) Effect of initial PFOA concentration (10–1000 μM) on the removal rate and adsorption capacity of PA6F membranes. (e) PFOA adsorption performance of PA6F membranes at different pH levels. (f) Desorption behavior of PA6F membranes after 168 h (7 days) in ultrapure water (UPW).

To investigate whether the adsorption behavior originated from the polymer itself or was primarily a result of nanofiber morphology, we conducted a comparative study using the same mass (10 mg) of PA6F in different physical forms: polymer powder (PA6F-p) and hot-pressed dense film (PA6F-h). Both the powder and hot-pressed film (Figure 3a) exhibited minimal adsorption during the initial 7 h, highlighting the importance of high surface area and accessible porosity provided by the electrospun nanofiber architecture.

In contrast, electrospun PA6 and PA66 nanofiber membranes, which share similar amide-containing backbones but lack the aromatic furan moiety, showed negligible adsorption throughout the entire 7 days, suggesting that neither the polyamide structure nor the nanofibrous morphology alone is sufficient for effective PFOA uptake, but rather the specific chemical structure of PA6F is essential.

Over extended time periods (up to 7 days), all three PA6F-based formats (nanofibers, powder, and hot-pressed) exhibited excellent adsorption performance (Figure 3b). The nanofiber membrane achieved the highest removal rate of 94.6%, while the powder and dense film reached 90.9% and 91.5%, respectively. These results confirm that PA6F intrinsically possesses a strong affinity for PFOA, and the nanofiber form enhances the kinetics by maximizing surface accessibility.

To further test the membrane’s application potential under dynamic conditions, a simple gravity-driven filtration apparatus was employed, with PA6F membranes placed between compartments. When PFOA solution was passed through the membrane driven by gravity, 54.3% of the PFOA was retained in a single pass (Figure 3c), indicating that the membrane could serve as an effective filtration medium in flow-through applications.

The effect of the initial PFOA concentration on the adsorption behavior of PA6F membranes was also evaluated (Figure 3d). When the initial PFOA concentration increased from 10 to 1000 μM, the removal rate gradually decreased from 94.6% to 24.3%, which is expected due to saturation of available adsorption sites. However, the adsorption capacity (mg g^–1^) increased accordingly from 3.92 to 100.64 mg g^–1^, following typical adsorption behavior driven by concentration gradients.^35^ At higher concentrations, more PFOA molecules are available for adsorption, leading to a higher total uptake. However, as binding sites on the membrane become saturated, the fraction of PFOA removed from the solution decreases, resulting in a lower removal rate despite an increase in absolute adsorption capacity.

The influence of solution pH (ranging from 4.4 to 9.8) on PFOA adsorption was examined by adjusting the pH of 10 μM PFOA solutions using HCl or NaOH. PA6F membranes exhibited consistently high adsorption efficiency across this range, with the highest removal rate observed near neutral pH (Figure 3e). PFOA has a reported p*K*_a_ of ∼3.8,^36^ meaning it exists predominantly in its anionic form (−COO^–^) under all tested conditions. Zeta potential measurements showed that the PA6F membrane is positively charged under acidic conditions and negatively charged under basic conditions. Thus, electrostatic attraction would be expected at low pH, while repulsion may occur at high pH. Despite these expected trends, PA6F membranes maintained strong adsorption capacity even at near-neutral and slightly basic pH, suggesting that electrostatic interactions are not the sole governing mechanism. Upon swelling, the membrane exposes polar functional groups that facilitate hydrogen bonding and dipolar interactions with PFOA molecules. These additional nonelectrostatic interactions help compensate for the reduced electrostatic driving force at higher pH, enabling efficient adsorption across a broad pH range.

To assess the retention strength of PFOA within the PA6F membrane, desorption experiments were performed by immersing PFOA-loaded membranes into fresh ultrapure water and monitoring the PFOA concentration over time (Figure 3f). Even after 7 days, only minimal PFOA release was observed, with the residual concentration remaining below 2%, indicating strong retention within the membrane. This suppressed desorption is attributed to a combination of strong polymer-PFOA interactions and physical confinement arising from hydration-induced matrix densification. These results indicate that the PA6F nanofiber membranes exhibit fast adsorption kinetics, high capacity, and stable performance for PFOA removal. This behavior can be attributed to the polymer’s distinctive molecular structure, polar surface chemistry, and ability to undergo swelling-induced densification. The interplay between chemical interactions and morphological responsiveness highlights the potential of PA6F as an effective material platform for capturing persistent organic pollutants.

To preliminarily assess the robustness of PA6F nanofibrous membranes under more realistic aqueous conditions, a mixed-contaminant adsorption experiment was conducted in which PFOA was copresent with methyl orange (MO), a commonly used anionic organic dye. MO was selected as a model cocontaminant due to its negative charge and aromatic character, which enable competitive interactions with polymer adsorption sites and are widely employed to probe coadsorbate effects in adsorption studies.^37,38^ As shown in Figures S9 and S10 and Table S5, Supporting Information, PA6F maintains substantial PFOA removal even in the presence of a competing anionic organic species, indicating that the adsorption–confinement mechanism is not readily disrupted by coadsorbates.

### 3.4 Mechanism Studies and Molecular Modeling

Molecular dynamics (MD) simulations indicate persistent proximity and multipoint contacts between PFOA and PA6F chains under hydrated conditions, supporting a cooperative adsorption and confinement mechanism rather than a single, specific binding interaction.

The distinctive swelling-shrinkage behavior of PA6F membranes in aqueous environments can be interpreted in terms of disruption and reorganization of intermolecular interactions within the polymer network. Water molecules act as both hydrogen bond donors and acceptors and, upon immersion, penetrate the membrane and interact with amide functionalities and polar furan moieties, while partially weakening existing intra- and interchain polymer interactions. This process leads to fiber swelling and softening. Subsequent elastic retraction of polymer chains, together with capillary-driven contraction within the porous network, results in macroscopic membrane shrinkage and matrix densification.

Regarding the PFOA adsorption mechanism, zeta potential measurements show that PA6F membranes carry a highly positive surface charge under acidic conditions (e.g., +40 mV at pH 3), suggesting that electrostatic attraction toward anionic PFOA (−COO^–^) is favored at low pH. However, experimental adsorption results demonstrate that PFOA uptake is maximized near neutral pH (∼7), indicating that electrostatic interactions alone are insufficient to account for the observed adsorption behavior.

Under the experimental pH conditions investigated, PFOA exists predominantly in its deprotonated carboxylate form (−COO^–^). Accordingly, hydrogen-bond-related interactions are more plausibly associated with the carboxylate oxygen atoms of PFOA acting as hydrogen bond acceptors, as well as with water-mediated hydrogen bonding within the hydrated polymer matrix, rather than involving a carboxylic proton. In addition, hydrophobic association between the perfluorinated carbon chain of PFOA and the polymer matrix is expected to contribute to adsorption. Although PA6F is a polar polyamide, the presence of semiaromatic furan units and aliphatic segments provide regions capable of accommodating hydrophobic interactions with fluorinated chains.

These chemical interactions operate in concert with hydration-induced matrix densification and physical confinement. Localized swelling during immersion may enhance the accessibility of interaction sites within the polymer network, while subsequent contraction restricts molecular mobility and effectively traps PFOA within the densified fiber matrix.

Atomistic MD simulations provide further insight into these cooperative effects. Representative simulation snapshots (Figure 4a–c and Supporting Information Video S1) show PFOA molecules remaining in close proximity to reorganized and locally collapsed PA6F chains over the course of the simulation, supporting a mechanism dominated by sustained proximity, multipoint interactions, and physical confinement rather than discrete stoichiometric complex formation. Radial distribution function analysis reveals a pronounced first coordination peak between PFOA and PA6F chains (Figure 4b), indicating preferential spatial association under hydrated conditions relative to bulk water.

**Figure 4:**
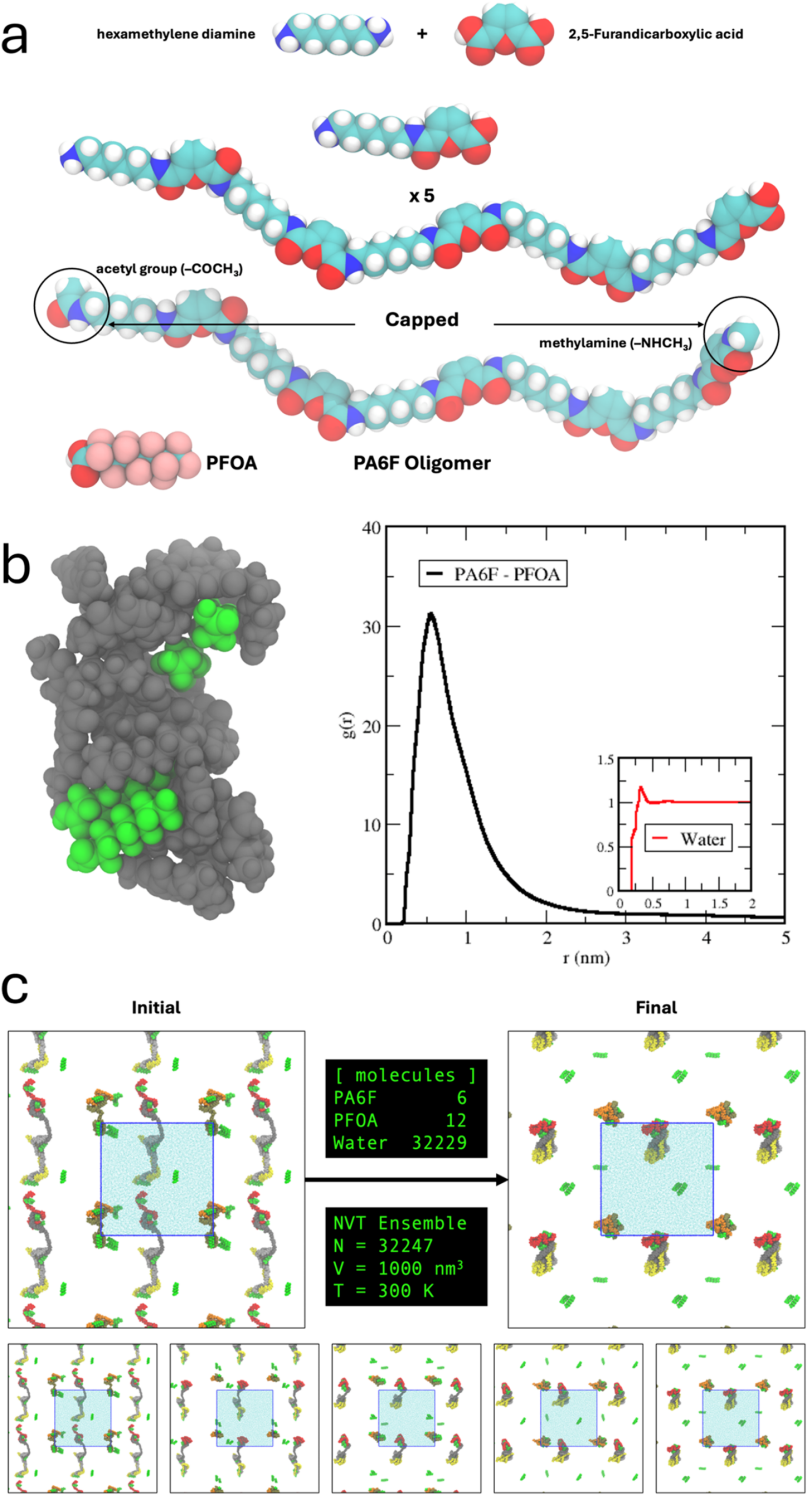
Molecular-level investigation of PFOA uptake. (a) Atomistic representation of the capped (PA6F) five oligomer and PFOA molecules used in simulations. (b) Radial distribution functions, *g*(*r*), describing the spatial correlation between PA6F and PFOA in water. The inset shows the corresponding radial distribution function for water as a reference; the inset shares the same axes as the main plot. (c) Representative simulation snapshots sequence of PFOA-polymer interactions in water.

### 3.5 Reusability via PFOA Thermal Treatment and Re-Electrospinning

Given the strong PFOA adsorption and retention ability of PA6F membranes, their potential for regeneration and reuse through thermal treatment was investigated. Thermogravimetric Analysis/Mass Spectrometry (TGA-MS) was employed to evaluate the thermal stability of PA6F membranes and to investigate whether the selective removal of the adsorbed PFOA could be achieved without degradation of the polymer.

The pristine PA6F membrane showed excellent thermal stability, with only a minor weight loss of ∼2.4% near 110 °C due to moisture evaporation, followed by a major degradation step between 360 and 450 °C (maximum rate at 447 °C). No fluorinated ion signals (*m*/*z* 69 or 100) were detected (Figure 5a).

**Figure 5:**
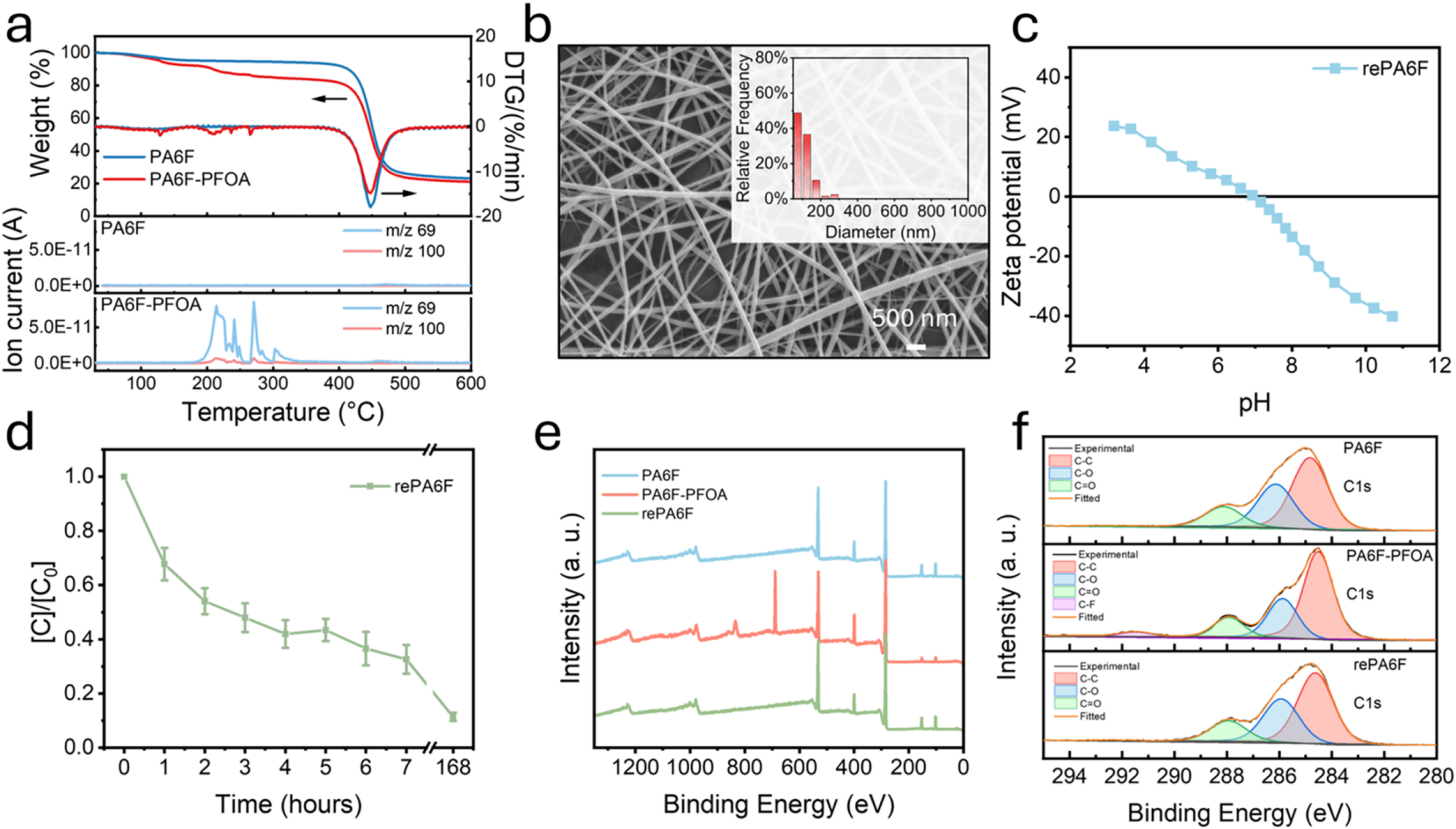
Thermal regeneration and reusability of PA6F nanofiber membranes. (a) TGA and MS analysis of pristine PA6F and PFOA-adsorbed PA6F (PA6F-PFOA) nanofiber membranes (after immersion in 10 mL of 2 mM PFOA solution). (b) SEM image of re-electrospun PA6F membrane (rePA6F). (c) Zeta potential profile of rePA6F membrane. (d) PFOA adsorption performance of rePA6F membrane. (e) XPS survey spectra and (f) high-resolution C 1s XPS spectra of PA6F, PA6F-PFOA, and rePA6F membranes.

In contrast, PA6F membranes loaded with PFOA showed a higher initial weight loss of 3.3%, indicating greater water retention due to membrane swelling and physical entrapment of water within its dense fiber network, consistent with FTIR evidence of hydrogen bonding interactions. For TGA-MS measurements, a higher PFOA concentration (2 mM in 10 mL solution, with 50 mg of membrane) was used to ensure sufficient PFOA uptake to enable clear thermal detection of its desorption. Under standard adsorption conditions (10 μM PFOA, 10 mg membrane), the total uptake is below 0.5% and is difficult to distinguish thermogravimetrically. In the high-loading condition, the PA6F-PFOA sample exhibited an additional weight loss of ∼9.7% in the 200–300 °C range, accompanied by a pronounced ion current at *m*/*z* 69 (CF_3_^+^), a characteristic PFOA fragment, together with a weaker signal at *m*/*z* 100 (C_2_F_4_^+^). These features evidence that the mass loss arises from the release of adsorbed PFOA, predominantly through desorption with limited decomposition. At higher temperatures (350–400 °C), both membranes displayed comparable polymer degradation without further fluorinated ion release. Collectively, these results demonstrate that PFOA can be effectively removed from PA6F membranes by moderate thermal treatment (∼240–300 °C), while the polymer remains structurally stable up to ∼400 °C, underscoring the feasibility of closed-loop regeneration.

To realize selective removal of PFOA via thermal treatment, PFOA-adsorbed PA6F membranes were thermally treated at 240 °C for 4 h under a nitrogen atmosphere in a tubular furnace. The resulting material was successfully redissolved in formic acid/DCM (1:1 v/v) and re-electrospun under the same optimized conditions. The regenerated nanofiber membrane, referred to as rePA6F, maintained a uniform morphology with an average fiber diameter of 104.54 ± 45.66 nm (Figure 5b), comparable to the original PA6F nanofibers, suggesting that the thermal treatment did not compromise the polymer’s processability or its ability to form nanofibrous structures. Furthermore, the zeta potential of rePA6F showed an isoelectric point close to pH 7 (Figure 5c), similar to that of PA6F after water exposure, indicating that the membrane’s surface charge properties were successfully restored after thermal treatment and re-electrospinning.

To evaluate the functional recovery of the regenerated membrane, the rePA6F was treated with a PFOA solution under the same conditions as previously. The rePA6F membrane achieved 67.4% removal within 7 h (Figure 5d) and reached 88.6% removal after 7 days (168 h), closely approaching the 93% removal rate of the original PA6F membrane. This result demonstrates that the adsorption performance of the material is largely retained after regeneration, validating the recyclability of PA6F through moderate heating and re-electrospinning.

X-ray photoelectron spectroscopy (XPS) further confirmed the successful removal of PFOA from the regenerated material. High-resolution XPS spectra of the C 1s regions showed clear C–F bonding contributions in the PFOA-adsorbed PA6F sample (Figure 5e,5f). In contrast, no C–F bond signal was detected following thermal treatment.

To further contextualize the performance of PA6F nanofibrous membranes, a comparison with representative PFOA adsorbent materials reported in the literature is provided in Table S6, Supporting Information. As summarized in the table, several emerging porous materials, such as MOFs and COFs, exhibit exceptionally high adsorption capacities (often exceeding 1000 mg g^–1^); however, these materials typically rely on fully synthetic frameworks, complex synthesis routes, and solvent-intensive regeneration processes that limit their practical scalability and sustainability.^39,40^

Conventional adsorbents, including activated carbon and ion-exchange resins, show moderate adsorption capacities (≈30 mg g^–1^) and are widely used in practice, yet they are predominantly fossil-based and use chemical regeneration, often generating secondary waste streams.^41,42^ In contrast, although the adsorption capacity of PA6F nanofibrous membranes is lower than that of some high-capacity porous adsorbents, PA6F offers a distinct combination of advantages that are rarely achieved simultaneously: a biobased polymer feedstock, operation in a solid membrane format, and a demonstrated closed-loop regeneration strategy based on mild thermal treatment followed by re-electrospinning.

In addition to demonstrating balanced adsorption performance, PA6F nanofiber membranes offer a distinct advantage over conventional membrane-based approaches. Unlike conventional reverse osmosis (RO) and nanofiltration (NF) membranes, which rely primarily on pressure-driven size exclusion and charge-based rejection and typically require high operating pressures and significant energy input, the PA6F nanofibrous membranes operate via a fundamentally different adsorption-based mechanism. As summarized in Table S7, Supporting Information, RO and NF processes generate concentrated brine streams that require further handling or disposal, whereas PA6F directly captures and immobilizes PFOA within the polymer matrix, avoiding secondary waste streams. The PA6F system enables regeneration through mild thermal treatment and potential material reuse, offering a closed-loop strategy with reduced energy demand and improved sustainability compared to conventional membrane technologies.

## 4 Conclusions

This work presents the first demonstration of manufacturing electrospun nanofiber membranes based on the biobased semiaromatic polyamide PA6F, synthesized from a renewable furanic monomer. The resulting membranes exhibit a unique nanofiber-swelling behavior in water, leading to macroscopic shrinkage and changes in surface properties, phenomena not observed in structurally similar polyamides, such as nylon-6 and nylon-66. When coupled with the polymer’s inherently polar amide and furan units, the membrane is able to achieve efficient and rapid adsorption of PFOA from water. As a result, the PA6F nanofibers demonstrate excellent performance for the adsorption of perfluorooctanoic acid (PFOA), achieving rapid removal (∼50% within 1 h) and high removal efficiency (∼94.6%).

Importantly, the nanofibrous membranes showed strong PFOA retention and were successfully regenerated via selective thermal treatment, removing PFOA without degrading the polymer. The recovered PA6F was re-electrospun into new membranes (rePA6F), which maintained both fiber morphology and adsorption capacity (∼88.6% removal after 7 days), demonstrating closed-loop recyclability.

Overall, this study demonstrates that PA6F nanofiber membranes are not only effective in capturing persistent organic pollutants, but also offer the advantages of renewability and reprocessability, which enables a closed-loop membrane regeneration strategy for sustainable water purification applications. This combination of features positions PA6F, and potentially other furanic polyamides, as promising candidates for the development of a new class of sustainable materials for advanced water treatment and other environmental applications.

## Supplementary Material





## Notes

Molecular dynamics simulation of water-mediated confinement and PFOA adsorption in PA6F (MP4)
Additional information on synthesis of PA6F; design of experiments (DOE) of electrospun nanofibers; preparation of PA6F hot-pressed film; LCMS measurement methods and calibration curve; FTIR spectra of PA6F powder and nanofiber membranes; SEM images; tensile properties of nanofiber membranes; adsorption under mixed-contaminant conditions; comparison of representative PFOA adsorbent materials and conventional RO/NF membranes (PDF)
