# Supplementary material for: Water-Induced Confinement of Perfluorinated Pollutants in Biobased Polyamide Nanofibrous Membranes
Source: ACS Appl Mater Interfaces. 2026 Mar 18;18(12):18368–79. doi: 10.1021/acsami.5c22145 (PMC13051448; doi:10.1021/acsami.5c22145)
Supplement: Supplementary file 2 [file am5c22145_si_002.pdf]

Supporting Information

Water-Induced Confinement of Perfluorinated Pollutants in Biobased Polyamide Nanofibrous Membranes

Xiang Ding[1,2], Muhammad Kamran[3], Garyfalia A. Zoumpouli[3,5], Guadalupe Jiménez-Serratos[4], Carmelo Herdes[3], Matthew G. Davidson[1,2], and Hannah S. Leese[1,2,3,]*

1. Department of Chemistry, University of Bath, Bath BA2 7AY, U.K.
2. Institute of Sustainability and Climate Change, University of Bath, Bath BA2 7AY, U.K.
3. Department of Chemical Engineering, University of Bath, Bath BA2 7AY, U.K.
4. The Hartree Centre, STFC Daresbury Laboratory, Warrington WA4 4AD, U.K.
5. School of Chemistry, University College Dublin, Dublin D04 V1W8, Ireland.

*corresponding author: h.s.leese@bath.ac.uk

Synthesis of poly(hexamethylene 2,5-furandicarboxylamide) (PA6F)

PA6F was synthesized following a previously reported two-step melt polymerization procedure.[1,2] Typically, dimethyl 2,5-furandicarboxylate (DMFDC, 0.2 mol) and hexamethylenediamine (HMDA, 0.2 mol) were charged into a reaction flask equipped with a mechanical stirrer and a nitrogen inlet. In the first stage, the mixture was heated at 65 °C under nitrogen atmosphere for 3 hours to form PA6F oligomers through transesterification and amidation reactions. Subsequently, the reaction mixture was gradually heated to 230 °C under reduced pressure (approximately 0.1 mbar) for an additional 5 hours, promoting polycondensation and achieving high molecular weight polymer chains. After cooling to room temperature, the resulting polymer was ground, purified by extraction with methanol to remove residual monomers, and subsequently dried under vacuum at 80 °C for 24 hours, yielding PA6F as a light-yellow solid powder.

Design of Experiments (DOE) of PA6F nanofibers

To identify the optimal electrospinning parameters for producing uniform, fine PA6F nanofibers, a Taguchi L_9 orthogonal array design was employed. Three process parameters were selected: applied voltage (15, 20, 25 kV), tip-to-collector distance (10, 15, 20 cm), and solution flow rate (2, 6, 10 μL/min), each tested at three levels (Table S1).

A "smaller-the-better" quality characteristic was applied to evaluate the experimental results and determine the optimal combination of electrospinning parameters for minimizing both the fiber diameter and its variation.[3] The signal-to-noise (S/N) ratio was calculated using the following equation:[4]

$$\frac{S}{N} = -10\log\left(\frac{1}{n}\sum_{i=1}^{n} {y_i}^2\right)$$

where S/N is the signal-to-noise ratio, y_i represents the measured fiber diameter for each run, and n is the number of observations. A higher S/N ratio indicates lower variance and better performance in achieving finer fibers.

Table S1. L_9 orthogonal design for selected factors with their levels.

**Run**	**Factors**		
	Voltage (kV)	Distance (cm)	Flow rate (μL/min)
**PA6F-1**	15	10	2
**PA6F-2**	15	15	6
**PA6F-3**	15	20	10
**PA6F-4**	20	10	6
**PA6F-5**	20	15	10
**PA6F-6**	20	20	2
**PA6F-7**	25	10	10
**PA6F-8**	25	15	2
**PA6F-9**	25	20	6

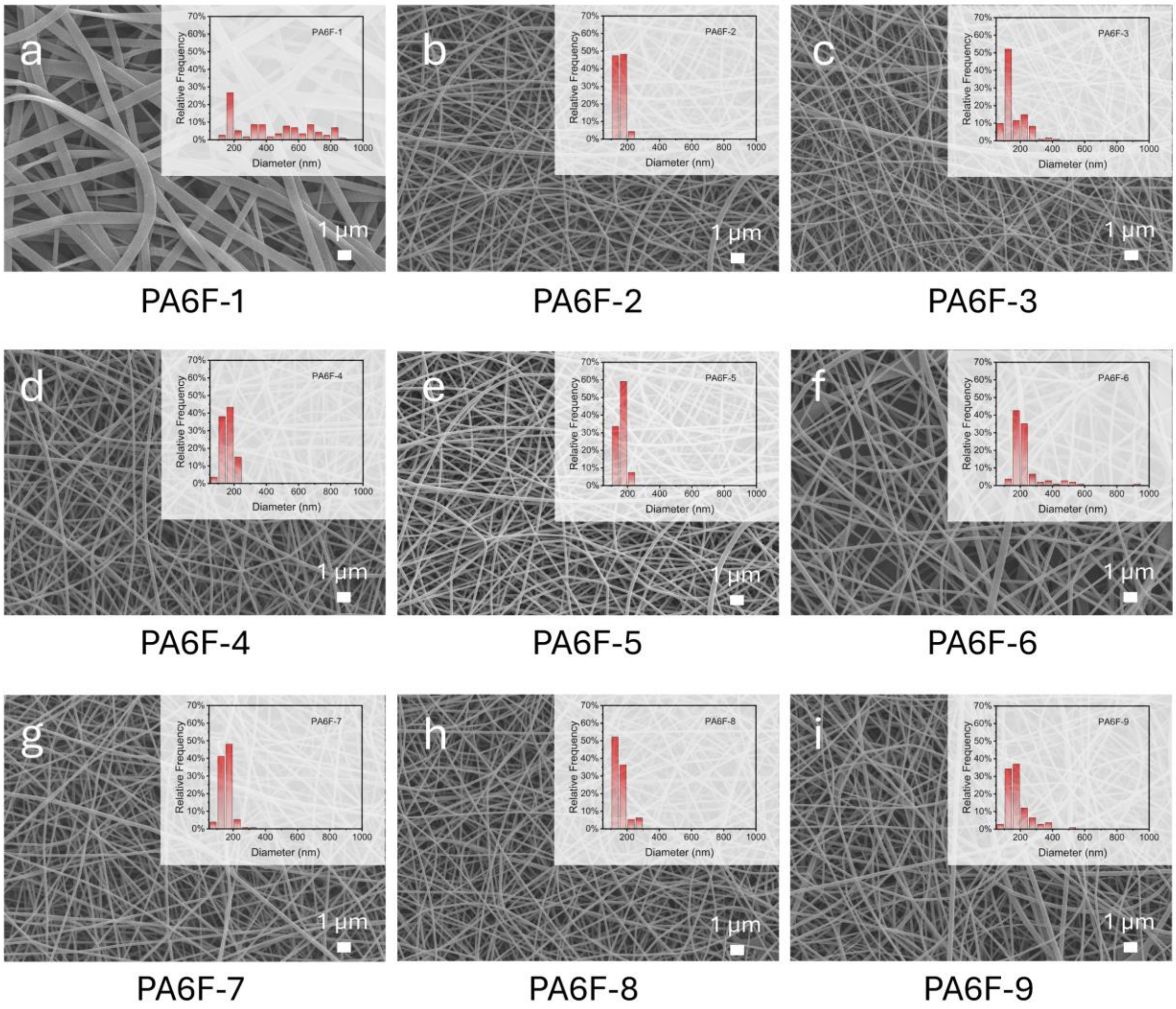

Figure S1. SEM images and corresponding fiber diameter distributions of electrospun PA6F nanofibers produced according to the experimental design shown in Table S1. (a-i) PA6F-1 to PA6F-9, respectively.

Table S2. PA6F nanofiber diameter with standard deviation and their corresponding signal-to-noise (S/N) ratios based on 'smaller the better'.

Run	Diameter (nm)	S/N values
**PA6F-1**	440.2 ± 247.5	-54.1
**PA6F-2**	152.4 ± 19.8	-43.7
**PA6F-3**	161.5 ± 66.1	-44.8
**PA6F-4**	158.7 ± 37.0	-44.2
**PA6F-5**	161.0 ± 26.0	-44.2
**PA6F-6**	234.6 ± 107.6	-48.2
**PA6F-7**	154.4 ± 34.7	-44.0
**PA6F-8**	158.2 ± 38.3	-44.2
**PA6F-9**	180.9 ± 70.9	-45.8

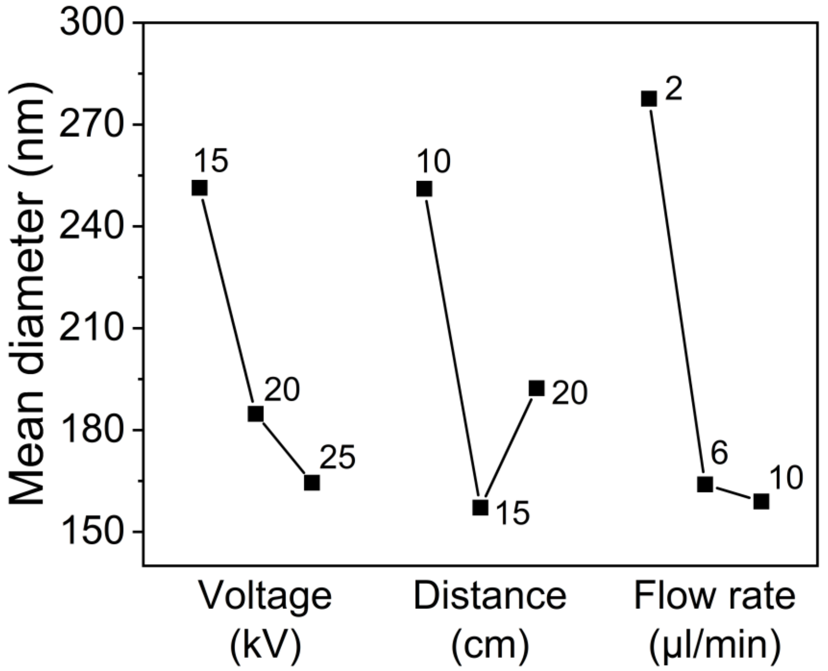

Figure S2. Main effect plots of each parameter on means of PA6F nanofiber diameter.

Table S3. Response table for means of fiber diameters and S/N values.

Run	Means of fiber diameters (nm)			Means of S/N values		
	Voltage	Distance	Flow rate	Voltage	Distance	Flow rate
**Level 1**	251.4	251.1	277.7	-49.1	-49.1	-49.6
**Level 2**	184.8	157.2	164.0	-45.5	-43.9	-44.3
**Level 3**	164.5	192.3	159.0	-44.3	-45.8	-44.0
**Range**	86.9	93.9	118.7	4.8	5.2	5.6
**Importance**	3	2	1	3	2	1
**Best level**	25kV	15cm	10μL/min			

The main effects plot (Figure S2) and response table (Table S3) indicate that flow rate had the most significant impact on fiber diameter, followed by distance and voltage, as reflected by their respective S/N ranges. Lower flow rates favored the formation of thinner fibers by reducing the volume of polymer delivered per unit time, thus improving jet stretching and solvent evaporation. An intermediate collector distance (15 cm) provided optimal fiber formation, likely due to balanced flight time for jet elongation and solvent removal. Voltage showed a weaker but still relevant influence, where the highest level (25 kV) supported finer jet formation without inducing instability.

The optimized parameter combination identified was 25 kV, 15 cm, and 10 μL/min, which yielded uniform nanofibers with a mean diameter of ~141 nm and minimal bead formation. These optimized fibers were used throughout the study for membrane fabrication and adsorption performance evaluation.

Electrospinning of nylon 6 (PA6) and nylon 66 (PA66) nanofiber membranes

Electrospun nanofiber membranes of nylon 6 (PA6) and nylon 66 (PA66) were prepared according to previously reported methods with minor modifications.[5,6] For PA6 nanofibers, nylon 6 polymer pellets were dissolved in a binary solvent mixture of formic acid (FA) and dichloromethane (DCM) at a ratio of 3:1 (v/v) to achieve a concentration of 20% (w/v). The PA6 polymer solution was electrospun at a flow rate of 4 μL/min, an applied voltage of 20 kV, and a spinneret-to-collector distance of 15 cm. The fibers were collected continuously onto an aluminum foil-covered rotating drum collector (200 rpm).

For PA66 nanofibers, nylon 66 pellets were dissolved similarly in FA/DCM (4:1, v/v) binary solvent mixture at a concentration of 10% (w/v). The electrospinning was carried out at a flow rate of 6 μL/min, an applied voltage of 20 kV, and a spinning distance of 15 cm under the same collector configuration. The obtained PA6 and PA66 nanofiber membranes were dried under vacuum at 50 °C overnight prior to further use.

Preparation of PA6F hot-pressed film

PA6F polymer powder was molded into dense films using a hot-pressing technique. Briefly, approximately 2 g of PA6F powder was placed into a rectangular metal mold and pressed at 80 °C under constant pressure for 10 minutes. After cooling to room temperature, a homogeneous film with an approximate thickness of 0.5 mm was obtained and subsequently stored in a desiccator until further testing.

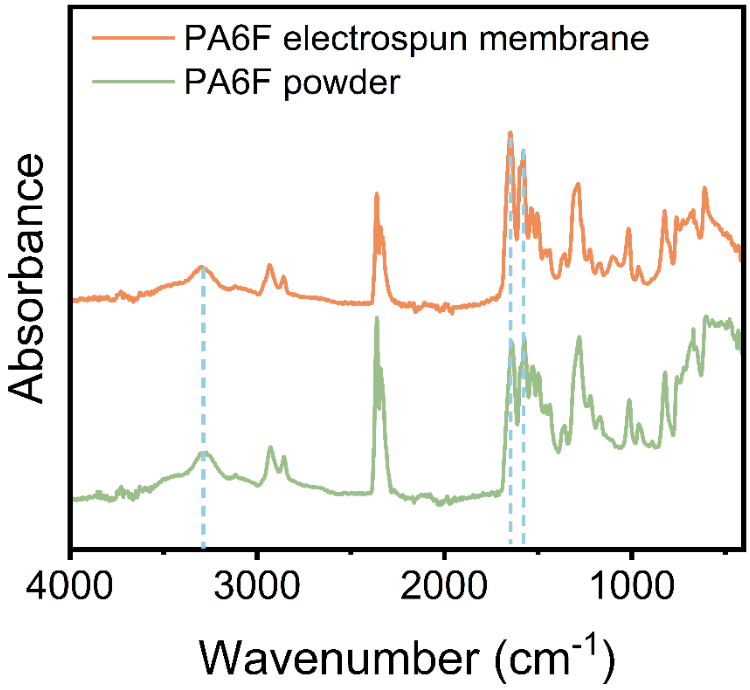

Figure S3. FTIR spectra of PA6F polymer powder and electrospun PA6F nanofiber membrane.

The characteristic N–H stretching vibration appeared at 3290–3294 cm^{-1}, confirming the presence of amide linkages. Peaks at 3116–3119 cm^{-1} corresponded to =C–H stretching vibrations of the furan ring, highlighting the incorporation of the aromatic heterocycle. The C–H asymmetric and symmetric stretching vibrations were observed at 2921–2932 cm^{-1} and 2851–2865 cm^{-1}, respectively, consistent with the aliphatic hexamethylene segments. The amide I band (C=O stretching) appeared at 1625–1646 cm^{-1}, while the amide II band (N–H bending) was found at 1524–1531 cm^{-1}. A signal at 1570–1573 cm^{-1} was attributed to aromatic C=C bending vibrations within the furan ring.[7] Both PA6F powder and electrospun membrane display consistent characteristic peaks, confirming that the chemical structure of the polymer remains unchanged during electrospinning.

Table S4. Porous properties of PA6F nanofibrous membranes

Condition	BET surface area ($m^2\ g^{-1}$)	MFP pore size (μm)
Dry	15.6	0.43
After 1 h water immersion	0.0095	—
After 24 h water immersion	Not detectable	—

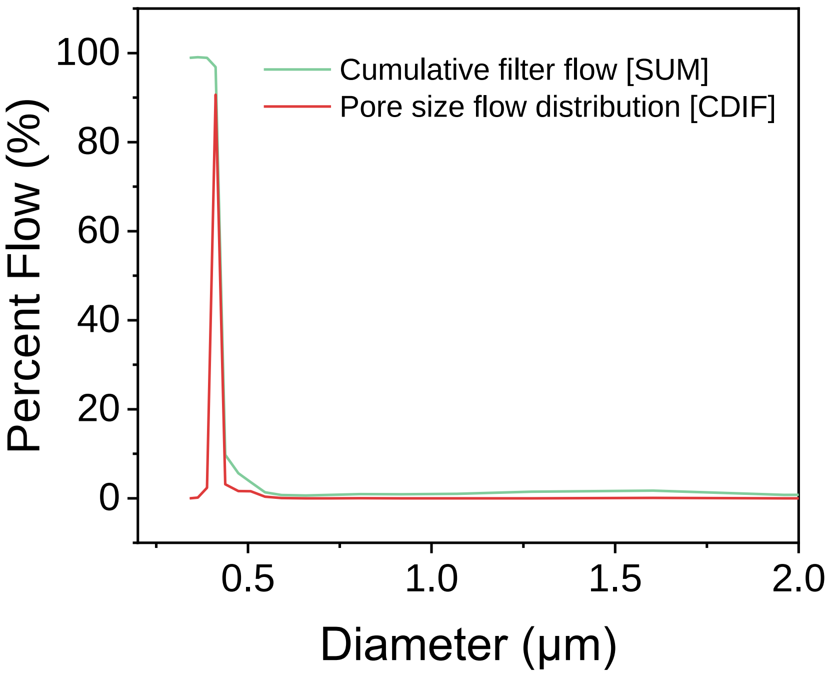

Figure S4. Pore size distribution of pristine PA6F nanofiber membrane.

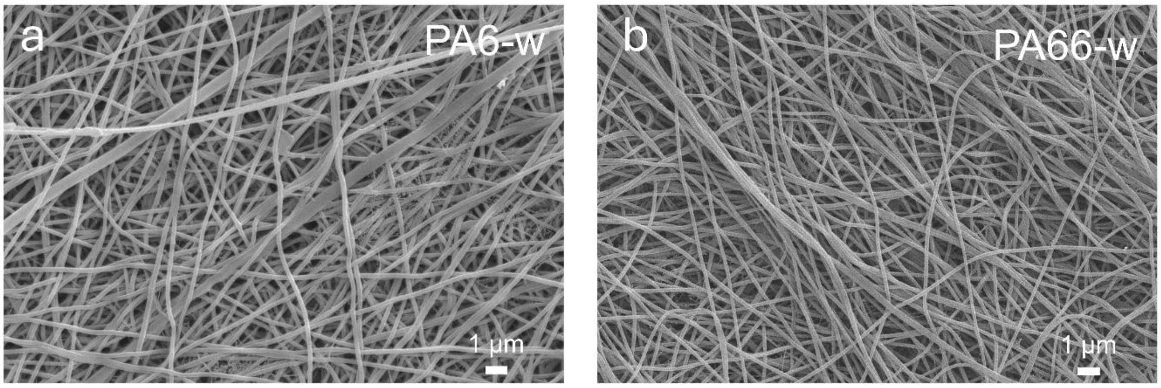

Figure S5. SEM images of (a) PA6 and (b) PA66 nanofibers after 1 h of water immersion.

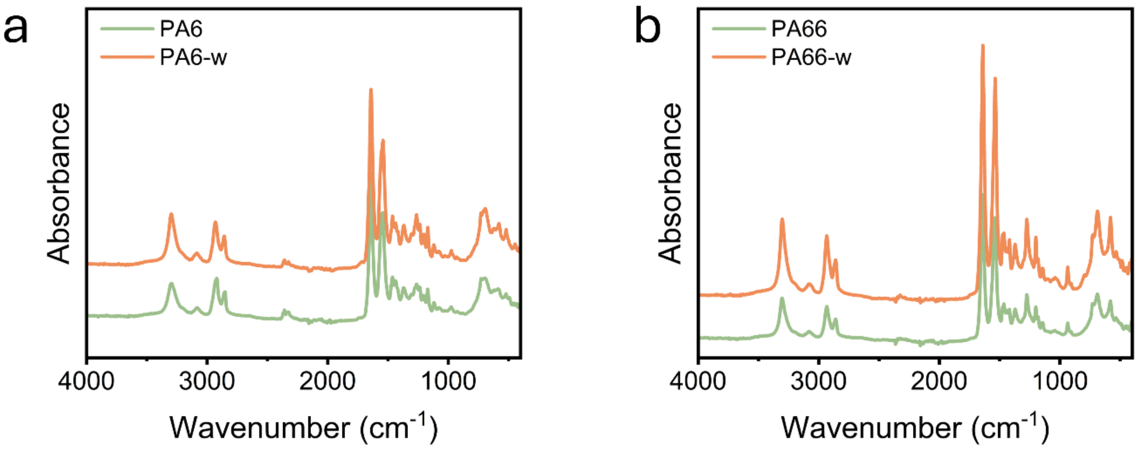

Figure S6. FTIR spectra of (a) PA6 and (b) PA66 nanofibers before and after 1 h of water immersion.

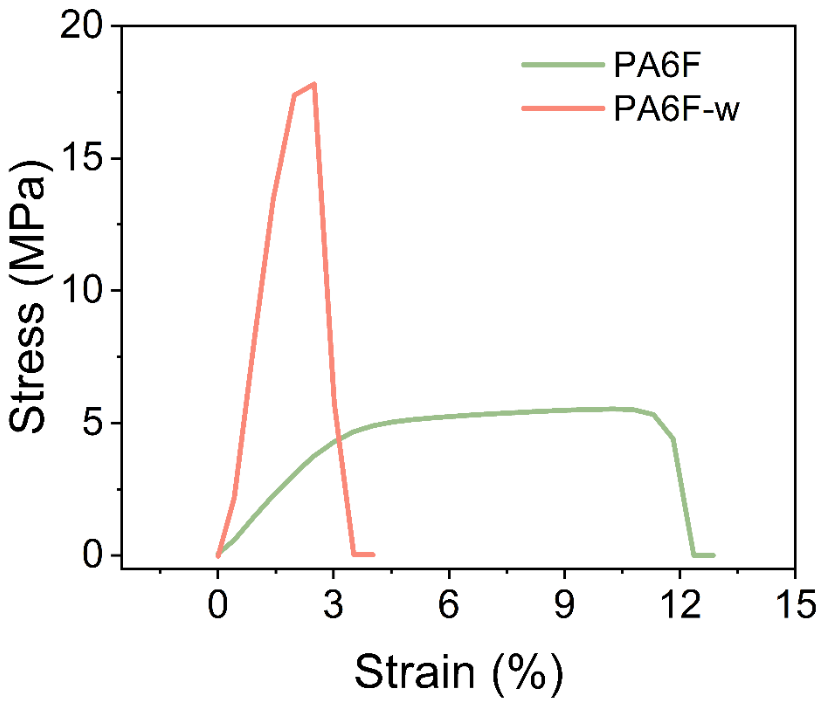

Figure S7. Tensile strength graph of PA6F nanofibers before and after 1 h of water immersion.

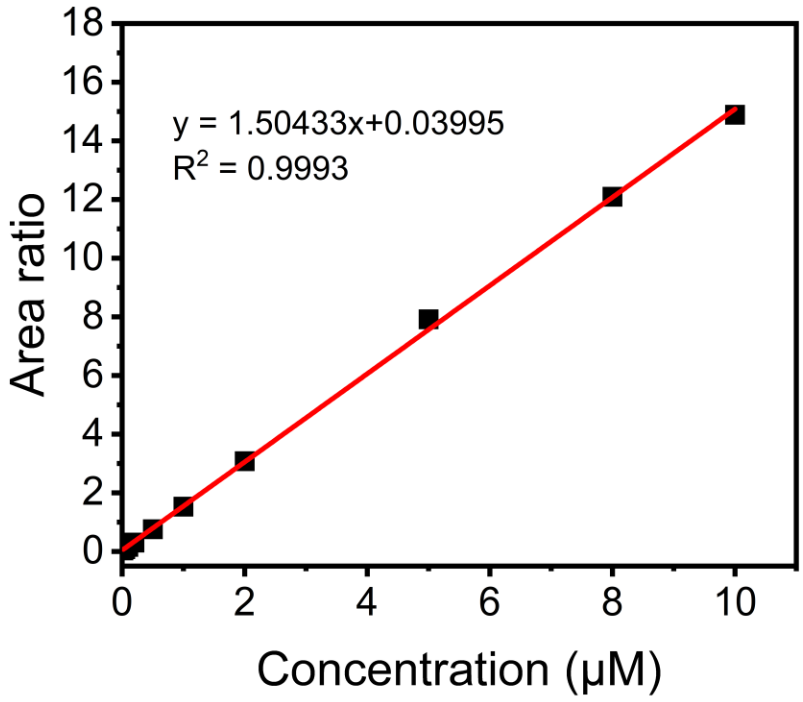

Figure S8. Calibration curve of PFOA using LCMS.

LC-MS measurement

Chromatographic separation was achieved using an RRHD Eclipse Plus C18 reverse-phase column (2.1 × 100 mm, 1.8 μm particle size), along with a C18 guard column (2.1 × 5 mm, 1.8 μm particle size), operated at 50 °C. The mobile phase consisted of 5 mM ammonium acetate in ultrapure water (A) and 5 mM ammonium acetate in methanol (B). A gradient elution was employed: 95% A for 1 min, decreasing linearly to 45% A at 4 min, then to 10% A at 13.5 min, and to 0% A at 14.5 min, held at 0% A for 3 min, and finally returning to 95% A with a total run time of 21 min. The flow rate was 0.35 mL min^{-1}, and the injection volume was 5 μL. Selected ion monitoring (SIM) mode was used for mass spectrometry. Each sample was spiked with an internal standard (stable isotope-labeled PFOA, Wellington Laboratories) at a concentration of 0.6 μM. The calibration curve for PFOA exhibited good linearity over the range of 0.02-10 μM (R^2 = 0.9993).

Adsorption under mixed-contaminant aqueous conditions

To preliminarily evaluate the adsorption behavior of PA6F nanofibrous membranes in the presence of co-contaminants, mixed-contaminant batch adsorption experiments were conducted using perfluorooctanoic acid (PFOA) and methyl orange (MO) as representative anionic organic pollutants. A mixed aqueous solution was prepared by combining 5 mL of a 10 mg/L MO solution with 5 mL of a 100 μM PFOA solution, yielding a final volume of 10 mL containing MO (5 mg/L) and PFOA (50 μM).

An electrospun PA6F nanofibrous membrane (10 mg) was immersed in the mixed solution and maintained under static conditions at room temperature for 7 days. After the adsorption period, the membrane was removed, and the supernatant solution was collected for analysis.

The residual concentration of MO was quantified by UV-vis spectroscopy. Absorbance spectra were recorded over the range of 200-800 nm, and the characteristic absorption peak at 464 nm was used for quantitative analysis. A calibration curve was constructed using standard MO solutions, and the peak height at 464 nm was used to determine the residual MO concentration (Figure S9). PFOA concentrations before and after adsorption were determined by LC-MS following the same analytical protocol described for the single-component adsorption experiments in the main text.

Following 7 days of contact with the PA6F nanofibrous membrane, the initially orange mixed solution became visually transparent (Figure S10), indicating substantial removal of the dye component. UV-vis spectra show a pronounced decrease in the characteristic MO absorption peak at 464 nm after adsorption (Figure S10). The increased absorbance in the UV region (~269 nm) is attributed to background UV absorption and/or scattering effects associated with prolonged membrane immersion. Quantitative analysis based on the calibration curve revealed that the MO concentration decreased from 5.0 mg/L to 0.12 mg/L, corresponding to a removal efficiency of approximately 97.6%.

In parallel, LC-MS measurements showed that the PFOA concentration decreased from an initial value of 50 μM to 7.4 μM after treatment, corresponding to an 85.2% removal efficiency. These results demonstrate that PA6F nanofibrous membranes retain substantial PFOA uptake even in the presence of a competing anionic organic contaminant.

The simultaneous removal of MO and PFOA suggests that adsorption in PA6F is governed by cooperative interaction mechanisms rather than a single, highly specific binding process. While MO is effectively removed through electrostatic interactions and π–π or hydrophobic associations with the polymer matrix, substantial PFOA removal is maintained despite the presence of a competing organic species. This behavior is consistent with the proposed adsorption mechanism involving a combination of electrostatic interactions, hydrogen bonding (direct and water-mediated), hydrophobic association, and physical confinement induced by water-triggered matrix densification.

It should be noted that this mixed-contaminant experiment serves as a proof-of-concept demonstration of adsorption robustness in the presence of a co-contaminant, rather than a comprehensive simulation of complex environmental water matrices. Systematic studies incorporating variations in ionic strength, dissolved organic matter, and additional PFAS species remain important directions for future investigation.

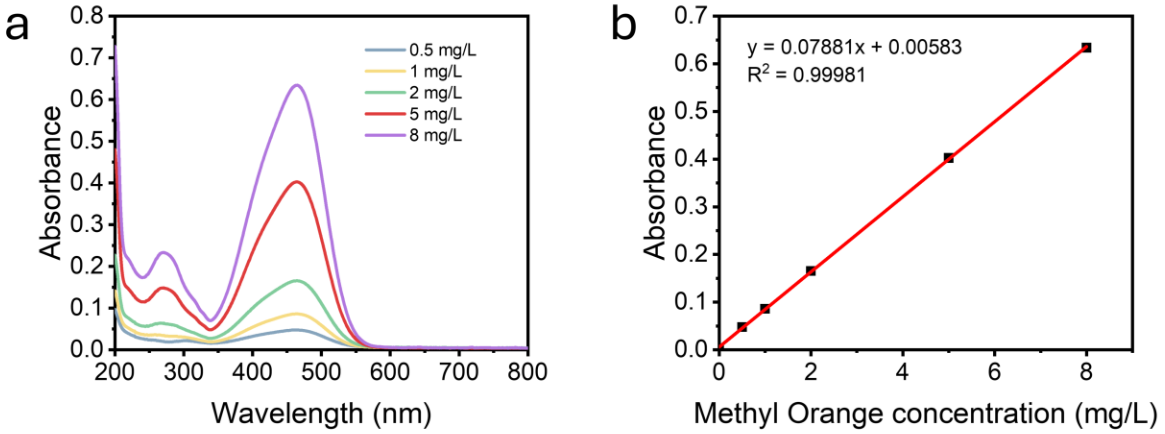

Figure S9. (a) UV-vis spectrum of methyl orange at different concentrations. (b) Calibration curve for methyl orange determined by UV-vis spectroscopy using the absorbance peak height at 464 nm.

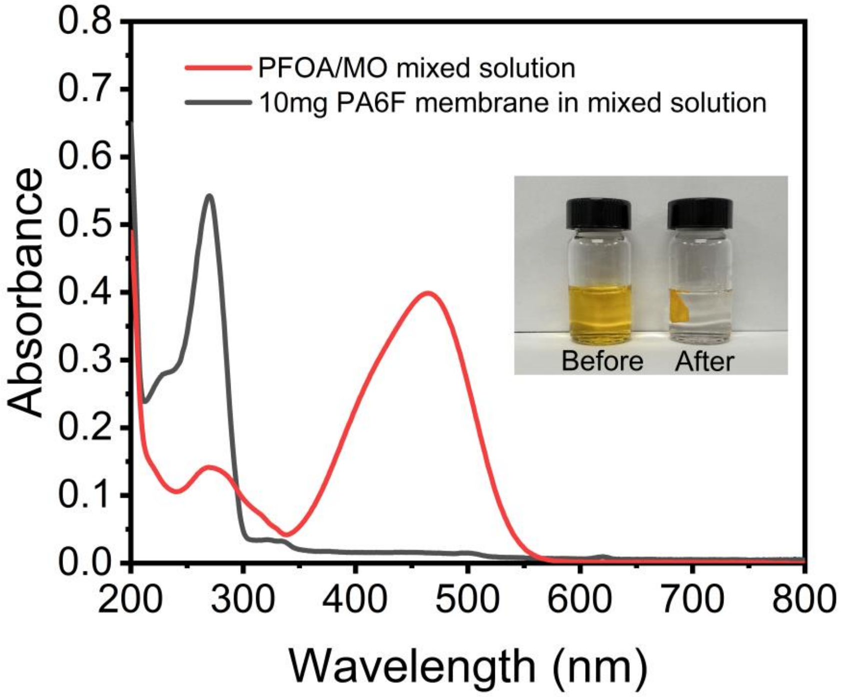

Figure S10. UV–vis absorption spectra of the mixed PFOA/MO solution before and after adsorption by PA6F nanofiber membranes. The inset shows photographs of the mixed solution before and after adsorption.

Table S5. Summary of mixed-contaminant adsorption performance.

**Contaminant**	**Initial concentration**	**Final concentration**	**Removal efficiency**
Methyl orange	5.0 mg/L	0.12 mg/L	97.6%
PFOA	50 μM	7.4 μM	85.2%

Table S6. Comparison of representative PFOA adsorbent materials reported in the literature.

Adsorbent type	Representative material	Adsorption capacity (mg g^{-1})	Adsorbent/Suspension (g/L)	Regeneration method	Sustainability / renewability	Reference
Carbon-based adsorbents	Activated carbon/Fe^0	33.11	1	Thermal / chemical regeneration	Fossil-based	[8]
	Carbon nanotube	2.76	0.25	-	Fossil-based	[9]
Ion-exchange resins	Anion-exchange resin IRA910	32.63	0.001	Chemical regeneration	Fossil-based	[10]
Graphene-based materials	GO $ZnFe_3O_4$/Chitosan	21.64	1	Solvent washing	Fossil-based	[11]
MOFs / COFs	UiO-66-type MOF	1178	0.5	Solvent extraction	Synthetic materials	[12]
	TG-PD COF	2600	0.4	Solvent extraction	Synthetic materials	[13]
Polymeric adsorbents	Molecularly imprinted polymers	5.45	0.2	Solvent extraction	Synthetic polymers	[14]
**Sustainable polyamide**	**PA6F nanofibrous membrane**	**3.92**	**1**	**Mild thermal treatment + re-electrospinning**	**Bio-based, renewable**	**This work**

Table S7. Comparison between PA6F nanofibrous membranes and conventional RO/NF membranes for PFAS removal.

Feature	PA6F nanofibrous membrane	Reverse osmosis (RO) membranes	Nanofiltration (NF) membranes
Primary removal mechanism	Adsorption + molecular confinement	Size exclusion + electrostatic repulsion	Size exclusion + charge effects
Operating pressure	Ambient / gravity-driven	High (typically 1-7 MPa)	Moderate (0.3-3 MPa)
Energy consumption	Low	High	Moderate
PFAS handling strategy	Capture and immobilization within polymer matrix	Rejection into concentrated brine	Partial rejection into concentrate
Secondary waste stream	No brine waste stream generated	Concentrated PFAS brine	Concentrated PFAS brine
Regeneration method	Mild thermal treatment (~240 °C) + re-electrospinning	Chemical cleaning / membrane replacement	Chemical cleaning / membrane replacement
Material renewability	Bio-based polymer (furan-derived)	Petroleum-based polymers	Petroleum-based polymers
Recyclability	Closed-loop reprocessing demonstrated	Limited	Limited
Reference	**This work**	[15,16]	[17]

Reference

(1) Kamran, M.; Davidson, M. G.; Tsanaktsis, V.; van Berkel, S.; de Vos, S. Structure-Property Insights of Semi-Aromatic Polyamides Based on Renewable Furanic Monomer and Aliphatic Diamines. *Eur. Polym. J.* **2022**, *178*, 111496. https://doi.org/10.1016/j.eurpolymj.2022.111496.

(2) Kamran, M.; Davidson, M. G.; de Vos, S.; Tsanaktsis, V.; Yeniad, B. Synthesis and Characterisation of Polyamides Based on 2,5-Furandicarboxylic Acid as a Sustainable Building Block for Engineering Plastics. *Polym. Chem.* **2022**, *13* (23), 3433–3443. https://doi.org/10.1039/D2PY00189F.

(3) Sorkhabi, T. S.; Samberan, M. F.; Ostrowski, K. A.; Zajdel, P.; Stempkowska, A.; Gawenda, T. Electrospinning of Poly (Acrylamide), Poly (Acrylic Acid) and Poly (Vinyl Alcohol) Nanofibers: Characterization and Optimization Study on the Effect of Different Parameters on Mean Diameter Using Taguchi Design of Experiment Method. *Materials*. 2022. https://doi.org/10.3390/ma15175876.

(4) İnanç Horuz, T.; Belibağlı, K. B. Production of Electrospun Gelatin Nanofibers: An Optimization Study by Using Taguchi's Methodology. *Mater. Res. Express* **2017**, *4* (1), 15023. https://doi.org/10.1088/2053-1591/aa57ea.

(5) Abbasi, A.; Nasef, M. M.; Takeshi, M.; Faridi-Majidi, R. Electrospinning of Nylon-6,6 Solutions into Nanofibers: Rheology and Morphology Relationships. *Chinese J. Polym. Sci.* **2014**, *32* (6), 793–804. https://doi.org/10.1007/s10118-014-1451-8.

(6) Matulevicius, J.; Kliucininkas, L.; Martuzevicius, D.; Krugly, E.; Tichonovas, M.; Baltrusaitis, J. Design and Characterization of Electrospun Polyamide Nanofiber Media for Air Filtration Applications. *J. Nanomater.* **2014**, *2014* (1), 859656. https://doi.org/10.1155/2014/859656.

(7) Jiang, Y.; Maniar, D.; Woortman, A. J. J.; Loos, K. Enzymatic Synthesis of 2,5-Furandicarboxylic Acid-Based Semi-Aromatic Polyamides: Enzymatic Polymerization Kinetics, Effect of Diamine Chain Length and Thermal Properties. *RSC Adv.* **2016**, *6* (72), 67941–67953. https://doi.org/10.1039/C6RA14585J.

(8) Gevaerd de Souza, N.; Parenky, A. C.; Nguyen, H. H.; Jeon, J.; Choi, H. Removal of Perfluoroalkyl and Polyfluoroalkyl Substances in Water and Water/Soil Slurry Using Fe0-Modified Reactive Activated Carbon Conjugated with Persulfate. *Water Environ. Res.* **2022**, *94* (1), e1671. https://doi.org/10.1002/wer.1671.

(9) Deng, S.; Zhang, Q.; Nie, Y.; Wei, H.; Wang, B.; Huang, J.; Yu, G.; Xing, B. Sorption Mechanisms of Perfluorinated Compounds on Carbon Nanotubes. *Environ. Pollut.* **2012**, *168*, 138–144. https://doi.org/10.1016/j.envpol.2012.03.048.

(10) Sukeesan, S.; Boontanon, S. K.; Boontanon, N.; Fujii, S. Regeneration of Ion-Exchange Resins and Granular Activated Carbon with the Sonochemical Technique for Enabling Adsorption of Aqueous Per- and Polyfluoroalkyl Substances. *IOP Conf. Ser. Earth Environ. Sci.* **2022**, *973* (1), 12004. https://doi.org/10.1088/1755-1315/973/1/012004.

(11) Elanchezhiyan, S. S. D.; Preethi, J.; Rathinam, K.; Njaramba, L. K.; Park, C. M. Synthesis of Magnetic Chitosan Biopolymeric Spheres and Their Adsorption Performances for PFOA and PFOS from Aqueous Environment. *Carbohydr. Polym.* **2021**, *267*, 118165. https://doi.org/10.1016/j.carbpol.2021.118165.

(12) Dalapati, R.; Shi, J.; Hunter, M.; Zang, L. Dual-Functional Metal–Organic Framework for Efficient Removal and Fluorescent Detection of Perfluorooctanoic Acid (PFOA) from Water. *J. Mater. Chem. C* **2025**, *13* (32), 16753–16762. https://doi.org/10.1039/D5TC01765C.

(13) Jrad, A.; Das, G.; Alkhatib, N.; Prakasam, T.; Benyettou, F.; Varghese, S.; Gándara, F.; Olson, M.; Kirmizialtin, S.; Trabolsi, A. Cationic Covalent Organic Framework for the Fluorescent Sensing and Cooperative Adsorption of Perfluorooctanoic Acid. *Nat. Commun.* **2024**, *15* (1), 10490. https://doi.org/10.1038/s41467-024-53945-4.

(14) Cao, F.; Wang, L.; Ren, X.; Sun, H. Synthesis of a Perfluorooctanoic Acid Molecularly Imprinted Polymer for the Selective Removal of Perfluorooctanoic Acid in an Aqueous Environment. *J. Appl. Polym. Sci.* **2016**, *133* (15). https://doi.org/10.1002/app.43192.

(15) Hara-Yamamura, H.; Inoue, K.; Matsumoto, T.; Honda, R.; Ninomiya, K.; Yamamura, H. Rejection of Perfluorooctanoic Acid (PFOA) and Perfluorooctane Sulfonate (PFOS) by Severely Chlorine Damaged RO Membranes with Different Salt Rejection Ratios. *Chem. Eng. J.* **2022**, *446*, 137398. https://doi.org/10.1016/j.cej.2022.137398.

(16) Mastropietro, T. F.; Bruno, R.; Pardo, E.; Armentano, D. Reverse Osmosis and Nanofiltration Membranes for Highly Efficient PFASs Removal: Overview, Challenges and Future Perspectives. *Dalt. Trans.* **2021**, *50* (16), 5398–5410. https://doi.org/10.1039/D1DT00360G.

(17) Chaudhary, M.; Sela-Adler, M.; Ronen, A.; Nir, O. Efficient PFOA Removal from Drinking Water by a Dual-Functional Mixed-Matrix-Composite Nanofiltration Membrane. *npj Clean Water* **2023**, *6* (1), 77. https://doi.org/10.1038/s41545-023-00286-2.
